# GPR35 Activation Reduces Ca^2+^ Transients and Contributes to the Kynurenic Acid-Dependent Reduction of Synaptic Activity at CA3-CA1 Synapses

**DOI:** 10.1371/journal.pone.0082180

**Published:** 2013-11-29

**Authors:** Rolando Berlinguer-Palmini, Alessio Masi, Roberto Narducci, Leonardo Cavone, Dario Maratea, Andrea Cozzi, Maria Sili, Flavio Moroni, Guido Mannaioni

**Affiliations:** 1 Department of Neuroscience, Psychology, Drug Research and Child Health, Section of Pharmacology and Toxicology, University of Florence, Florence, Italy; 2 Department of Health Science, Section of Clinical Pharmacology and Oncology, University of Florence, Florence, Italy; Goethe University Frankfurt, Germany

## Abstract

Limited information is available on the brain expression and role of GPR35, a Gi/o coupled receptor activated by kynurenic acid (KYNA). In mouse cultured astrocytes, we detected GPR35 transcript using RT-PCR and we found that KYNA (0.1 to 100 µM) decreased forskolin (FRSK)-induced cAMP production (p<0.05). Both CID2745687 (3 µM, CID), a recently described GPR35 antagonist, and GPR35 gene silencing significantly prevented the action of KYNA on FRSK-induced cAMP production. In these cultures, we then evaluated whether GPR35 activation was able to modulate intracellular Ca^2+^ concentration ([Ca^2+^]i ) and [Ca^2+^]i fluxes. We found that both KYNA and zaprinast, a phosphodiesterase (PDE) inhibitor and GPR35 agonist, did not modify either basal or peaks of [Ca^2+^]i induced by challenging the cells with ATP (30 µM). However, the [Ca^2+^]i plateau phase following peak was significantly attenuated by these compounds in a store-operated Ca^2+^ channel (SOC)-independent manner. The activation of GPR35 by KYNA and zaprinast was also studied at the CA3-CA1 synapse in the rat hippocampus. Evoked excitatory post synaptic currents (eEPSCs) were recorded from CA1 pyramidal neurons in acute brain slices. The action of KYNA on GPR35 was pharmacologically isolated by using NMDA and α7 nicotinic receptor blockers and resulted in a significant reduction of eEPSC amplitude. This effect was prevented in the presence of CID. Moreover, zaprinast reduced eEPSC amplitude in a PDE5- and cGMP-independent mechanism, thus suggesting that glutamatergic transmission in this area is modulated by GPR35. In conclusion, GPR35 is expressed in cultured astrocytes and its activation modulates cAMP production and [Ca^2+^]i. GPR35 activation may contribute to KYNA effects on the previously reported decrease of brain extracellular glutamate levels and reduction of excitatory transmission.

## Introduction

The G protein coupled receptor 35 (GPR35) was identified approximately 15 years ago [1], but its endogenous ligand as well as its role both in physiology and pathology are still not clear [2]. However, it is clearly demonstrated that GPR35 is expressed in the immune and gastro-intestinal systems, dorsal root ganglia (DRG), spinal cord, brain and cerebellum [3–5] and that zaprinast, a well-known cGMP PDE inhibitor [4], and kynurenic acid (KYNA) [5], a tryptophan metabolite, may activate this receptor. We have been particularly interested in studying the action of KYNA because of its ability to interact with different targets [6,7] and its proposed involvement in schizophrenia [8,9], cerebral ischemia [10] and degenerative neurological disorders [11]. We assumed that KYNA is the endogenous ligand for this receptor. However, the apparent low affinity of KYNA for the human form of the receptor and findings that other endogenous compounds such as lysophosphatidic acid may activate GPR35 with relatively high affinity [12] questioned this assumption [13]. A number of compounds such as pamoic acid [14], cromolyn disodium [15] and tyrphostin analogs have been recently described as GPR35 agonists [16,17].

KYNA has a number of other targets in the brain: it is a potent antagonist of the glycine allosteric site on the NMDA receptor complex and for several years it was assumed that the interaction between KYNA and the NMDA receptor could have a physiological role in brain function [18]. It has also been demonstrated that KYNA antagonizes α7 nicotinic receptors that are mostly located on pre-synaptic terminals [19] and it has been proposed that the reduced levels of glutamate in brain extracellular spaces found in KYNA treated animals are due to inhibition of these receptors. However, KYNA affinity for α7 nicotinic receptor is still rather low (µM) and certainly not in the range of the concentrations able to reduce glutamate release (low nM). Furthermore, other α7 nicotine receptor antagonists have some, but not all the actions of KYNA on excitatory transmitter release [20]. Thus, the reduction of glutamate concentration in the extracellular spaces cannot be exclusively ascribed to KYNA interaction with α7 nicotinic receptors and GPR35 remains one of the possible KYNA targets.

In previous studies, we reported that GPR35 is abundantly expressed in the DRG and the spinal cord of the rodents and that experimental elevation of KYNA concentration in the blood or brain significantly decreased glutamate extracellular levels in the nervous tissue and reduced inflammatory pain [21]. Since zaprinast (and other GPR35 agonists) had actions comparable with those of KYNA and the maximal effects of KYNA and zaprinast were not additive, we proposed that GPR35 activation was one of the mechanisms whereby KYNA reduced glutamate concentrations in brain extracellular levels and this could significantly decrease pain-activated neurotransmission [6,21].

In the present studies we firstly focused our attention on cultured astrocytes and we studied the effects of KYNA and zapinast on GPR35 activation, cAMP accumulation and calcium transients. A reduced intracellular cAMP concentration has been shown to modulate intracellular Ca^2+^ regulatory mechanisms in non-excitable cells [22]. We found that KYNA and zaprinast reduced FRSK-induced accumulation of cAMP and changed the shape of intracellular Ca^2+^ transients in astrocytes. Since it is widely accepted that astrocytic G-protein coupled receptors can tune synaptic transmission by modulating glutamate release in the synaptic cleft [23], we then investigated, in brain slices, the effects of KYNA and zaprinast on excitatory synaptic transmission. The results suggest that KYNA-induced activation of astrocytic GPR35 could contribute to the inhibitory action exerted by this tryptophan metabolite on excitatory synaptic function.

## Methods

### Ethical Statement

All animal manipulations were carried out according to the European Community guidelines for animal care (DL 116/92, application of the European Communities Council Directive 86/609/EEC). Formal approval to conduct the experiments described has been obtained from Italian Ministry of Health, according to DL 116/92. All efforts were made to minimize animal sufferings and to use only the number of animals necessary to produce reliable scientific data. No alternatives to animal experimentation are available for this type of experiments.

### Materials

6-Cyano-7-nitroquinoxaline-2,3-dione (CNQX), D(-)-2-amino-5-phosphonopentanoic acid (D-APV or DL-APV used at 50 or 100 µM, respectively), methyl-lycaconitine (MLA), gabazine, bicuculline, zaprinast sildenafil, MRS 1845 and Rp-8-Br-PET-cGMPs were obtained from Tocris (Bristol, UK). QX 314 was from Alomone laboratories (Jerusalem, Israel). Kynurenic acid (KYNA) and 3-isobutyl-1-methylxanthine (IBMX) were from Sigma-Aldrich (Milano, Italy). Methyl-5-[(tert-butylcarbamothioylhydrazinylidene)methyl]-1-(2,4-difluorophenyl)pyrazole-4-carboxylate (CID) was from Ryan Scientific (Mt Pleasant, SC, USA). Tissue culture reagents were obtained from Gibco-BRL (San Giuliano Milanese, MI, Italy) and ICN Pharmaceuticals (Opera, Milano, Italy). Unless otherwise declared, all cell culture media were purchased from Sigma-Aldrich.

### Preparation of mouse Dorsal Root Ganglia (DRG) and cortical astrocytes

DRG were prepared from neonatal mice (P7-P14) as described previously [24]. Cultured astrocytes were prepared as described previously [23]. Briefly, cerebral cortices from P0–P3 mice were dissected free of adherent meninges, minced and dissociated into a single cell suspension by trituration through a Pasteur pipette. Cells were grown in Dulbecco's modified Eagle's medium (DMEM; Gibco, cat. no. 11960-044) supplemented with 25 mM glucose, 10% heat-inactivated horse serum, 10% heat-inactivated fetal bovine serum, 2 mM glutamine and 1000 units/ml penicillin–streptomycin. Cultures were maintained at 37°C in a humidified atmosphere with 5% CO_2_. These cultures contain a high percentage of viable astrocytes (>90%), as previously demonstrated with by means of glial fibrillary acidic protein (GFAP) immunostaining [23].

### GPR35 detection by semi-quantitative and real time RT-PCR

RNA from DRG and astrocytic cultures was prepared using miRNeasy mini spin columns (Qiagen, Milano, Italy). cDNA was synthesized using equal amounts of RNA in each reaction (iScript^TM^ cDNA Synthesis Kit BIO-RAD, Milano, Italy). GPR35 mRNA levels were determined by semi-quantitative PCR performed with a Biometra thermal cycler (Gottingen, Germany), with the following amplification protocol:94°, 56°, 72°, (25 cycles), using the following primer pair: 5’-GGGGTACTGGCTCTCCCTAC-3’ and 5’-CCCAAGAGTCAACGTGCTTT-3’ (Integrated DNA Technologies, Leuven, Belgium). The ribosomal RNA 18S was amplified as an internal control. For real time PCR, RNA was extracted and reverse-transcribed as described above. The same primer pair was used and PCR was performed wih the Sybr Green kit (Qiagen) in a Rotorgene 3000 cycler system (Corbett Research, Australia).

#### GPR35 silencing in cultured astrocytes

Small interfering RNAs (siRNAs) used for GPR35 silencing in cultured astrocytes were purchased from Qiagen (Mm_Gpr35_10 siRNA, FlexiTube siRNA). The sequence of negative control siRNA (non silencing) was 5′-UUCUCCGAACGUGUCACGU-3′ (Qiagen). RNA was dissolved in the accompanying buffer and then in serum-free, oligofectamine-containing medium according to the manufacturer's instruction (Invitrogen, San Giuliano Milanese, Italy). Cells at 40-50% confluence were exposed to siRNA for 4 h at 37°C, and then an appropriate volume of DMEM plus serum was added to restore normal serum concentration (10%). After 48 h; astrocytes were processed for cAMP detection experiments (below) [21].. Effective GPR35 mRNA silencing was assessed with real-time PCR as described above.

### Determination of cAMP levels in cultured astrocytes

After ten days of culture in 24 well-plates, astrocytes were incubated with MEM/HEPES 10 mM for 10 min and then stimulated for 30 min with FRSK 10 μM in presence of IBMX 100 μM. KYNA or zaprinast were added in MEM containing HEPES. GPR35 antagonist (CID) was added 15 minutes prior to KYNA application. Reaction was stopped with lysis buffer from the Promega cAMP Glo assay kit and the plate shaken for 45 min at room temperature. cAMP level determination was performed with a VICTOR plate reader (Perkin Elmer).

#### Imaging of fluo-3 fluorescence in cultured astrocytes

Cultured astrocytes were incubated in a solution containing (mM): 150 NaCl, 10 HEPES, 3 KCl, 2 CaCl_2_, 1 MgCl_2_, 10 glucose (pH adjusted to 7.3) at 37°C for 30 min with the acetoxymethyl (AM) ester of fluo-3 (fluo-3 AM, 10 μg/ml; Molecular Probes, Milano, Italy). To aid solubilisation of fluo-3 AM in aqueous medium, we added pluronic F-127 (1 mM; Molecular Probes). The dye was then allowed to de-esterify for 30 min at room temperature. Coverslips containing fluo-3-loaded cells were subsequently transferred to a continuously perfused (2 ml/min) microscope stage for imaging. Images were visualized with a 20X/0.5W Fluor objective (Nikon) and acquired every 2 to 30 seconds accordingly to the temporal resolution needed. Exposure time was set to 25 to 50 ms and excitation was provided by 450-490 nm filtered light from a mercury lamp. Fluorescence was detected through a band pass filter (510-560 nm) with a Photometrics Coolsnap HP Camera set at -20°C. Fluorescence intensity was measured in cell bodies using Imaging Workbench 5 software (Indec BioSystem) and expressed as the ratio of (F - F_0_)/F_0_, where *F*_0_ is the fluorescence intensity before drug treatment. All measurements were corrected for the background fluorescence. Increases in fluorescence ratio greater than 0.2 were considered to be significant changes. Two ATP applications were performed in order to obtain an internal control. Drugs were applied before the second ATP response. The first peak obtained was then normalized to 100% and the area under the curve (AUC) of the second ATP response was calculated in control and in the presence of different drugs in a time period from 20 s to 70 s after the IP3-induced peak. The KYNA effect on capacitative calcium entry (CCE) was measured and compared to the one produced by the CCE selective blocker MRS 1845 after adding different concentrations of the drug to a calcium free solution where previous addition of the Sarco/Endoplasmatic Reticulum Calcium ATPase (SERCA) blocker thapsigargin had completely depleted the astrocytes calcium internal stores [25]. A rapid change from 0 mM Ca^2+^ solution to 2 mM Ca^2+^ solution produced an increase in cytosolic Ca^2+^ concentration due to CCE [26]. All experiments were performed at room temperature (20–23°C). 

#### Preparation of acute hippocampal slices and electrophysiological recordings

Preparation of hippocampal slices was carried out as previously described [23,27]. Young rats (Sprague-Dawley, age P14-P20) were deeply anesthetized with isoflurane and killed by decapitation. The brain was rapidly removed and submerged in an ice-cold artificial cerebrospinal fluid (ACSF) with the following composition (mM): 130 NaCl, 24 NaHCO_3_, 3.5 KCl, 1.25 NaH_2_PO_4_, 1 CaCl_2_, 3 MgSO_4_ and 10 glucose saturated with 95% O_2_/5% CO_2_, at pH 7.4. The hemisected brain was glued onto the stage of a vibrating micro-tome (Vibratome 1000s, Leica) and sections of 300 µm thickness were cut and stored in an incubation chamber at room temperature for about 1 h before use. The University of Florence Institutional Animal Care and Use Committee (IACUC) approved all procedures. Conventional visually-guided whole-cell patch recordings were obtained from CA1 pyramidal neurons in voltage clamp configuration using a Multiclamp 200 B (Molecular Devices, Sunnyvale, CA, USA) and a pipette with a resistance of 5-7 MΩ. The standard recording solution was composed of (mM): 130 NaCl, 24 NaHCO_3_, 3.5 KCl, 1.25 NaH_2_PO_4_, 1.5 CaCl_2_, 1.5 MgSO_4_ and 10 glucose saturated with 95% O_2_/5% CO_2_, at pH 7.4. All neurons included in this study had a resting membrane potential below –55 mV and an access resistance in the range of 10-20 MΩ that showed only minimal variations during the recordings included in this study. Recordings were filtered at 5 kHz and digitized at 20 KHz using a Digidata 1322A A/D board. All data were acquired, stored and analyzed on a PC using the pCLAMP, Origin and Graphpad Prism software (Molecular Devices, Sunnyvale, CA, USA and Microcal Software, Northampton, MA, USA, respectively). Drugs were administered by addition to the superfusing medium and were applied for a sufficient period to allow their full equilibration. All the data were collected at room temperature (23-26°C).

#### Measurement of evoked EPSCs (eEPSCs)

For recording of eEPSCs, electrodes were filled with (in mM) 140 K-gluconate, 10 HEPES, 7 NaCl, 4 Mg-ATP and 0.3 Na_3_-GTP. EPSCs were evoked from a holding potential of –60 mV by stimulation at a frequency of 0.06 Hz [28]. Stimuli were delivered through a bipolar stimulating electrode (240 µm spacing, FHC Inc., Bowdoinham, ME, USA) placed in the stratum radiatum within 100 µm of the patched cell. To avoid recurrent excitation, CA3 pyramidal layer was removed with a scalpel blade. 10 µM gabazine was used to abrogate GABA_A_- mediated inhibitory synaptic activity. D-APV (50 µM) and MLA (10-100 nM) were pre- and co-applied with KYNA in order to occlude the effects of KYNA on NMDA and α7 nicotinic receptors, respectively. In some experiments, GPR35 antagonist CID (10 µM) was included in the mix.

#### Statistics

Pooled data throughout the paper are presented as mean ± standard error (SEM) of *n* independent experiments (in parentheses in bar graphs). Sigmoidal dose-response curve (variable slope) was used to fit KYNA induced reduction of FRSK-induced cAMP production at different concentration. Unless otherwise specified, statistical difference between means is assessed with a two-sample Student t-Test for unpaired data (GraphPad Prism 5.0). Significance at the p < 0.05, 0.01 and 0.001 level is indicated with *, **, ***, respectively in figures. When traces are shown, they are intended to represent typical observations. Graphs, histograms and fittings were generated in GraphPad Prism 5.0.

## Results

### Identification of GPR35 in astrocytic cultures

**Figure 1:**
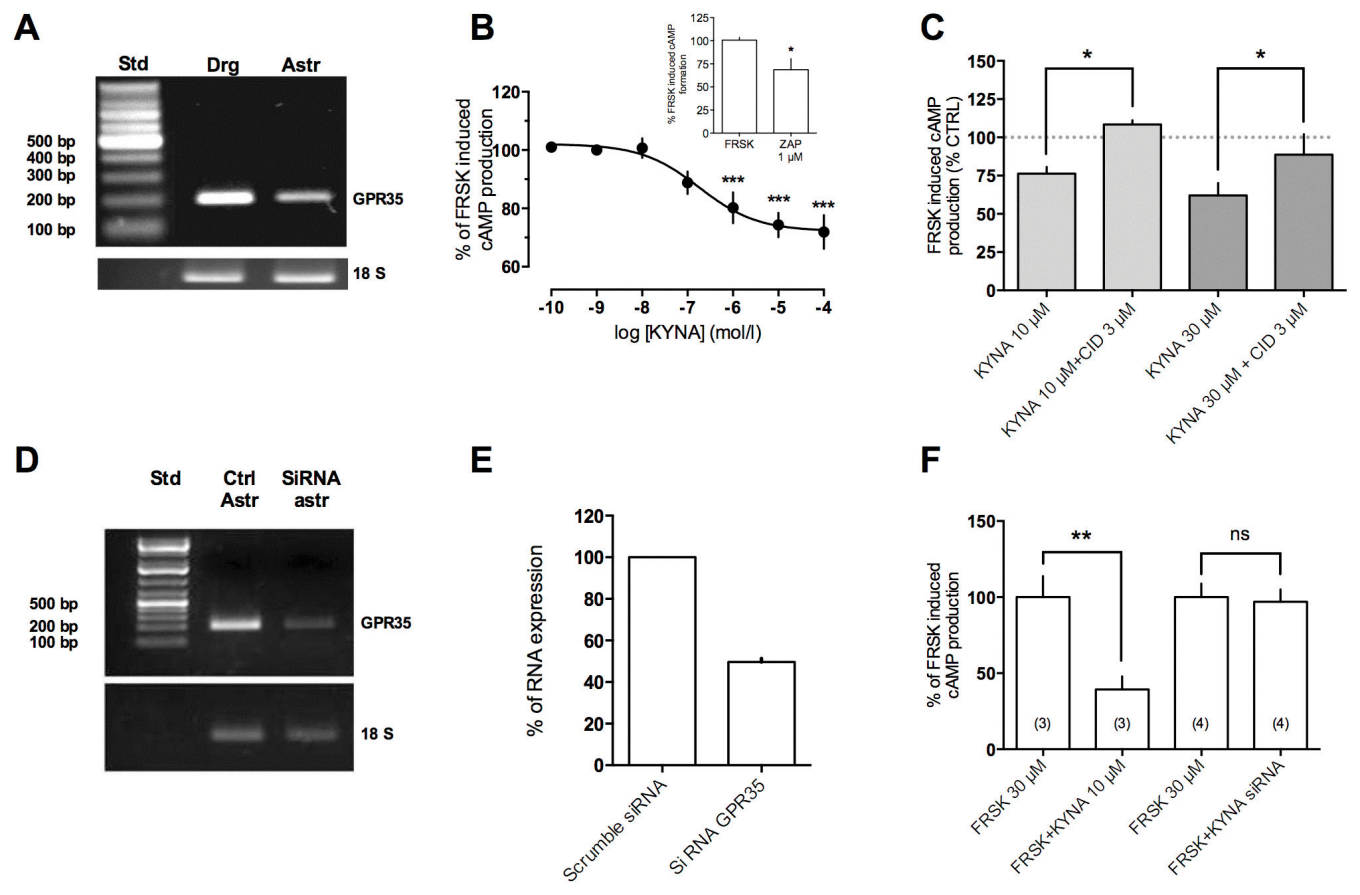
GPR35 is expressed in mouse cortical astrocytes and its activation provokes a decrease of FRSK-induced cAMP production. **A**) GPR35 transcripts were detected in cultured mouse astrocytes and in DRG after 10 days of in vitro cultures by RT-PCR. Ribosomal 18S RNA was amplified as an internal control. PCR products were analyzed by means of agarose gel electrophoresis. **B**) Concentration-response curve of KYNA on FRSK-activated cAMP formation in mouse astrocytic culture. Inset: zaprinast (1 µM), a different GPR35 agonist, induces a decrease of FRSK-activated cAMP formation. **C**) CID, prevents KYNA effects on FRSK-activated cAMP formation. **D**, **E** and **F**) GPR35 mRNA silencing reduces RNA expression and abolishes KYNA (10 µM) effects on FRSK-activated cAMP formation.

For the determination of GPR35 expression in cortical astrocytic cultures, we employed a semi-quantitative RT-PCR analysis. Figure 1A shows that a PCR product of the predicted size of 230 bp was amplified using cDNA generated from cultured astrocytes and DRG, a preparation rich in this receptor. Using immunohistochemistry, we previously reported that astrocytes express the protein in the cytosol and membranes, but not in the nucleus [29]. We then tested the effects of the putative GPR35 agonists KYNA and zaprinast on FRSK-induced increase of cAMP levels in these cells. This procedure has been widely used in our laboratory to study the action of Gi/o coupled receptors [30,31]. We found that both KYNA and zaprinast (at low concentrations) significantly reduced FRSK-induced cAMP accumulation (Figure 1B and inset). Interestingly, CID, a compound reported to be able to antagonize KYNA on mouse GPR35 [14], prevented the effects of KYNA on FRSK-induced cAMP formation (Figure 1C). Finally, using siRNA, we were able to significantly reduce GPR35 transcript levels (Figure 1D) with a transcript decrease of about 50% compared to scrambled siRNA (Figure 1E) and prevent KYNA-induced reduction of FRSK-dependent cAMP formation (Figure 1F).

### GPR35 and [Ca^2+^]i in astrocytes

**Figure 2:**
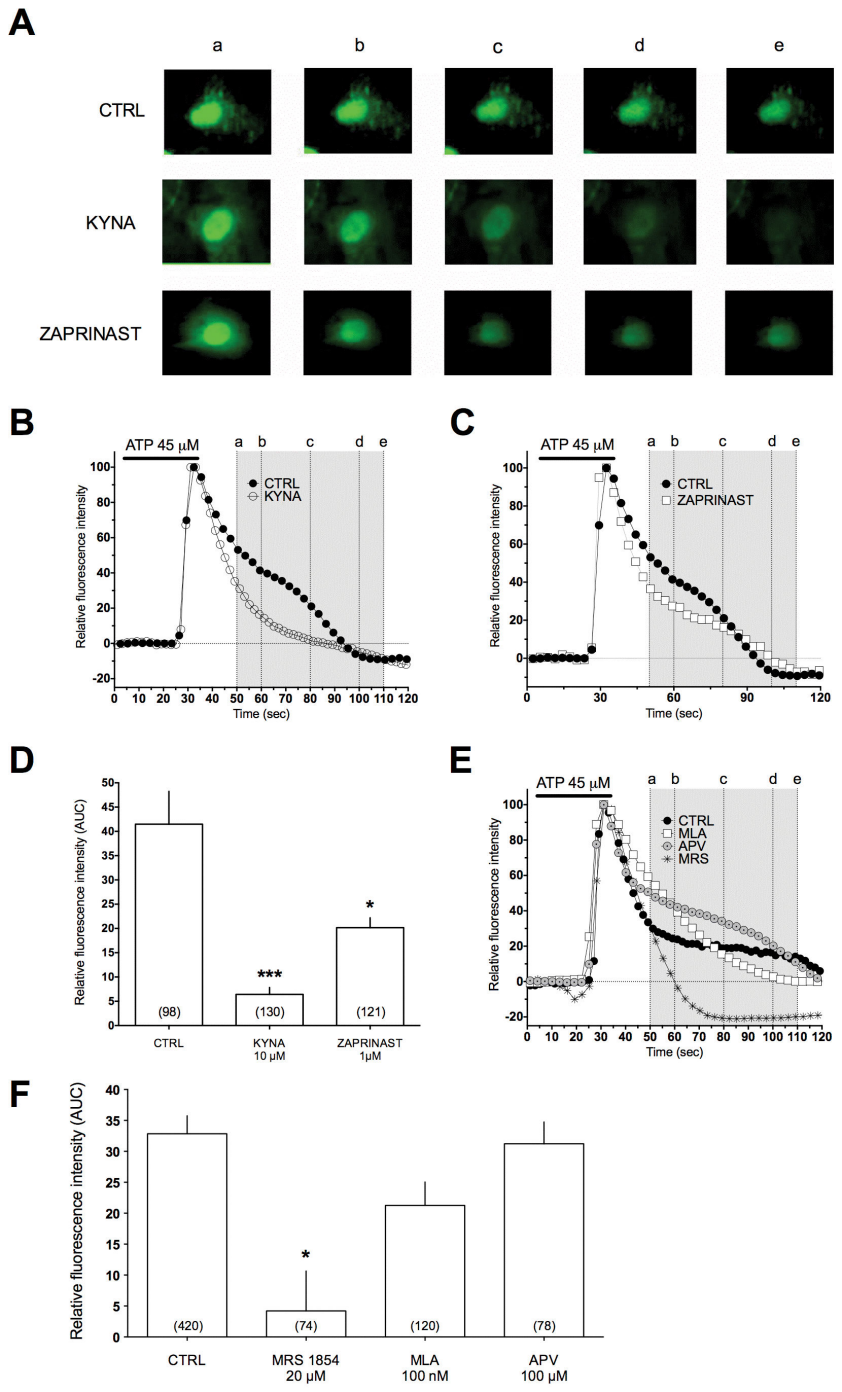
KYNA and zaprinast decrease the [Ca^2+^]i plateau phase following an IP3-mediated stimulus in mouse cortical astrocytes. **A**) A typical example of KYNA and zaprinast-induced decrease in somatic fluo-3 fluorescence time course measured in three different astrocytes in cultures: control (*upper panels*), in the presence of 10 μM KYNA (*middle panels*) or zaprinast 1 µM (*lower panels*). **B** and **C**) Time course of KYNA and zaprinast effects on fluo-3 fluorescence in a single astrocyte in culture. KYNA and zaprinast were applied 5 min before the recording was started. **D**) Area under the curve (AUC) of fluorescence intensity plot following an IP3 induced stimulus in control, KYNA and zaprinast, respectively. **E**) Time course of MRS-1845, D-APV (50 µM) and MLA (100 nM) effects on fluo-3 fluorescence in a single astrocyte in cultures. **F**) AUC following an IP3-induced stimulus in control, MRS-1845, D-APV and MLA. Area was calculated from 20 to 70 seconds after the ATP-induced fluorescence peak.

Activation of Gi protein-coupled receptors in astrocytes may reduce cAMP signaling pathways and modulate intracellular Ca^2+^ waves through a number of molecular mechanisms [32–34]. Agonist-induced Ca^2+^ signaling events in astrocytes typically consist of two phases: release of Ca^2+^ from internal stores (mainly the endoplasmic reticulum), which leads to a second phase of sustained Ca^2+^ entry across the plasma membrane. Therefore, we studied if and how GPR35 agonists could modify calcium waves induced by exposing cultured astrocytes to ATP (30 µM), which releases Ca^2+^ from internal stores by activating purinergic adenosine receptors ^2+^[32]. Figure 2A and 2B shows the effects of ATP on [Ca^2+^]i and the modulation of this effect in the presence of the GPR35 agonist KYNA. GPR35 activation decreases the plateau phase of the increase of [Ca^2+^]i following an IP3 mediated stimulus (ATP 30 µM) (Figure 2A, B and C). Interestingly, also zaprinast induced a decrease of [Ca^2+^]i plateau phase similar to the one obtained with KYNA, thus strengthening the proposition of a GPR35-mediated activation of this effect (Figure 2 C and D).

One of the mechanisms controlling the duration of the plateau phase of the waves is the opening of the so called “store operated calcium channels” after the IP3-induced Ca^2+^ release from the stores. This influx has also been named capacitative Ca^2+^ entry and in cultured cerebellar astrocytes is modulated by cAMP intracellular concentrations [22]. It is possible to reduce this influx with MRS-1845, and Figure 2 E and F show that the compound has a non-quantitative action comparable to that of KYNA. In order to rule out the possibility that the measeured effect is due to antagonism of NMDA or on α7 nicotinic receptors we also tested D-APV (100 µM) and MLA (100 nM) which antagonize NMDA or α7 nicotinic receptor, respectively. Figure 2E and 2F show that neither NMDA nor α7 nicotinic receptors antagonists modify calcium waves in astrocytes.

**Figure 3:**
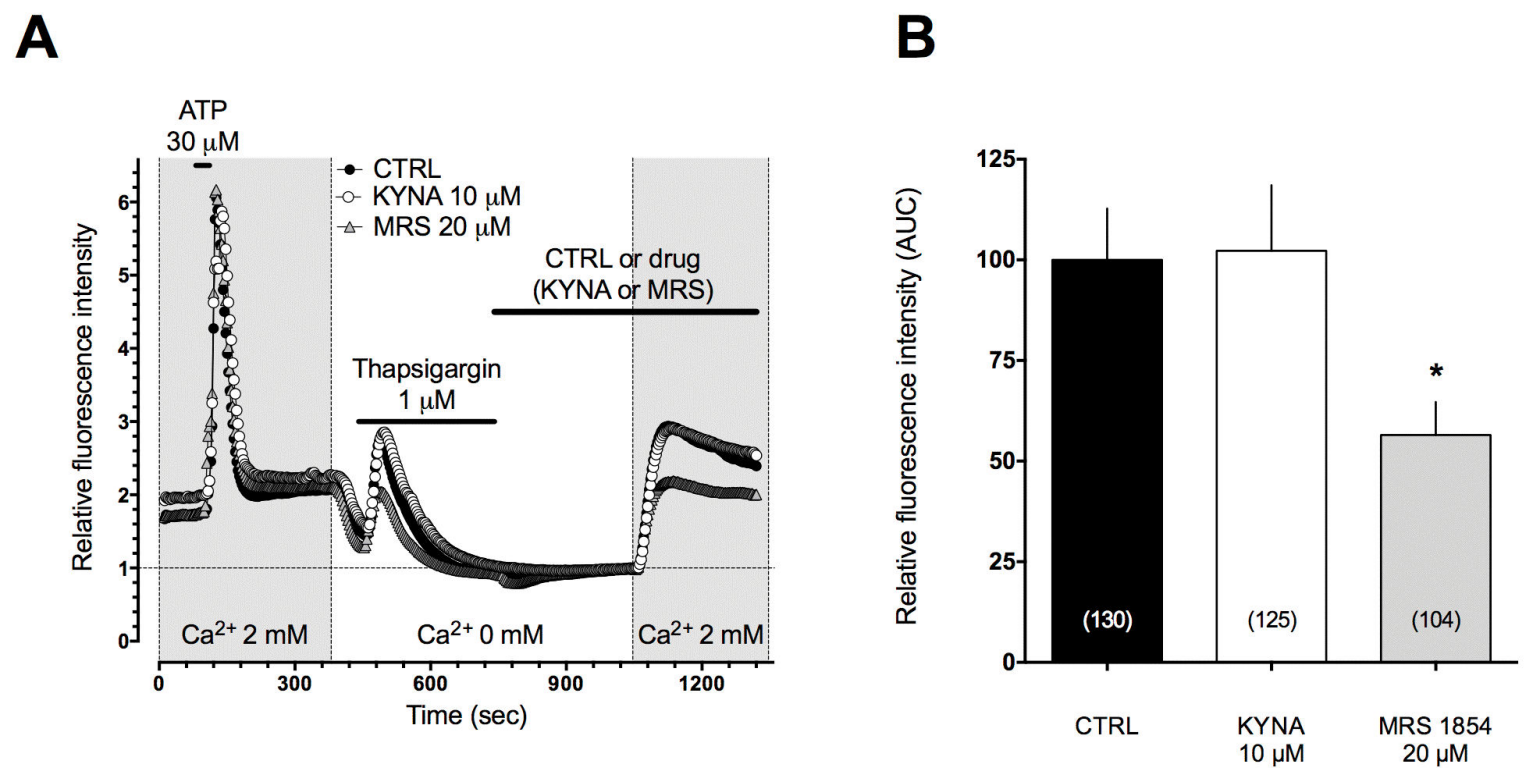
KYNA-induced decrease of [Ca^2+^]i plateau phase following an IP3-mediated stimulus is CCE-independent. **A**) Time course of Ca^2+^ fluxes following an IP3–mediated stimulus (external Ca^2+^ concentration, [Ca^2+^]e, 2 mM), a depletion of internal Ca^2+^ stores induced by thapsigargin in 0 mM [Ca^2+^]e and subsequent stores refilling (CCE) induced by restoring 2 mM [Ca^2+^]e . **B**) AUC following reinstatement of 2 mM [Ca^2+^]e showing the modulation of CCE by the selective SOC blocker MRS -1845 but not by KYNA 10 µM.

Since cAMP has been reported to increase capacitative calcium entry (CCE) and to modulate the length of the plateau phase of the Ca^2+^ signaling in astrocytes [22] and the actions of MRS-1845, an inhibitor of this influx, resemble those of KYNA and zaprinast, we used a specific protocol to investigate whether GPR35 activation with KYNA could reduce this calcium influx. Figure 3 reports that in astrocytes exposed to nominally Ca^2+^ free medium thapsigargin depletes Ca^2+^ stores passively by virtue of its ability to inhibit SERCA pumps on the endoplasmic reticulum and the ensuing entry of Ca^2+^ is therefore assumed to be the very definition of CCE [25,26]. The actions of appropriate concentrations of KYNA or of MRS1845 on these channels are rather different: MRS inhibits while KYNA does not affect this influx. These experiments rule out the possibility that GPR35 activation and the resulting reduced cAMP in the cells modify the plateau phase of calcium signaling by inhibiting SOCs.

### GPR35 activation reduces synaptic transmission at CA3-CA1 synapses

**Figure 4:**
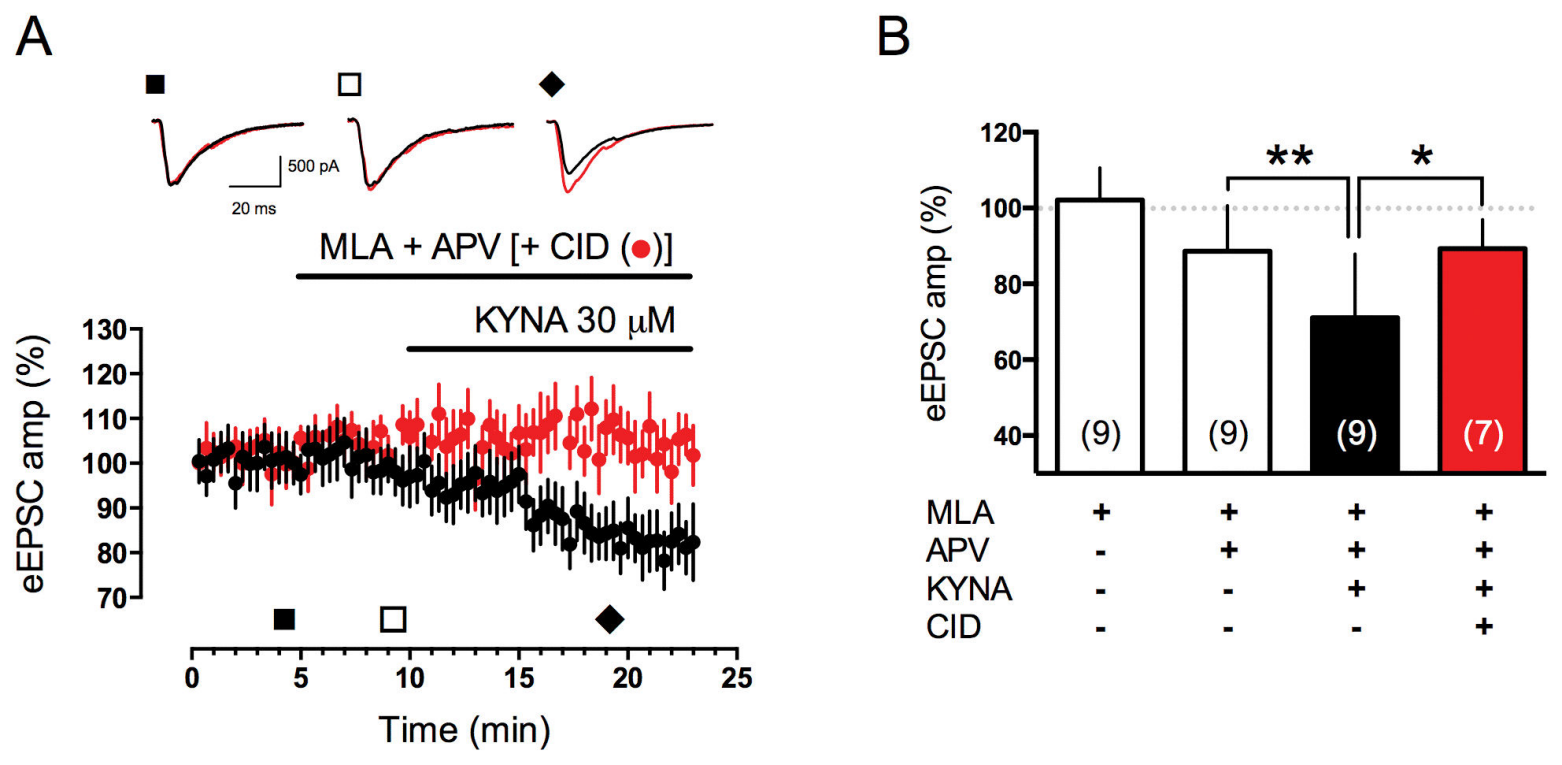
KYNA reduces eEPSCs amplitude at CA3-CA1 synapse in a GPR35 dependent manner. **A**) Time course of KYNA-induced decrease in eEPSC amplitude, normalized to pre-application values, in the presence of D-APV and MLA (50 µM and 100 nM, respectively; black dots), or D-APV, MLA and CID (10 µM; red dots). Sample traces are shown on top. **B**) Bar graph shows maximal effect of KYNA and its antagonism by CID. The effect of KYNA was statistically significant compared to pre-application level (third vs second column, One-Way ANOVA for repeated measures, followed by Tukey’s post hoc test). Preincubation with CID was able to prevent the effect of KYNA (fourth vs third column, two-way t-test for unpaired sets of data).

**Figure 5:**
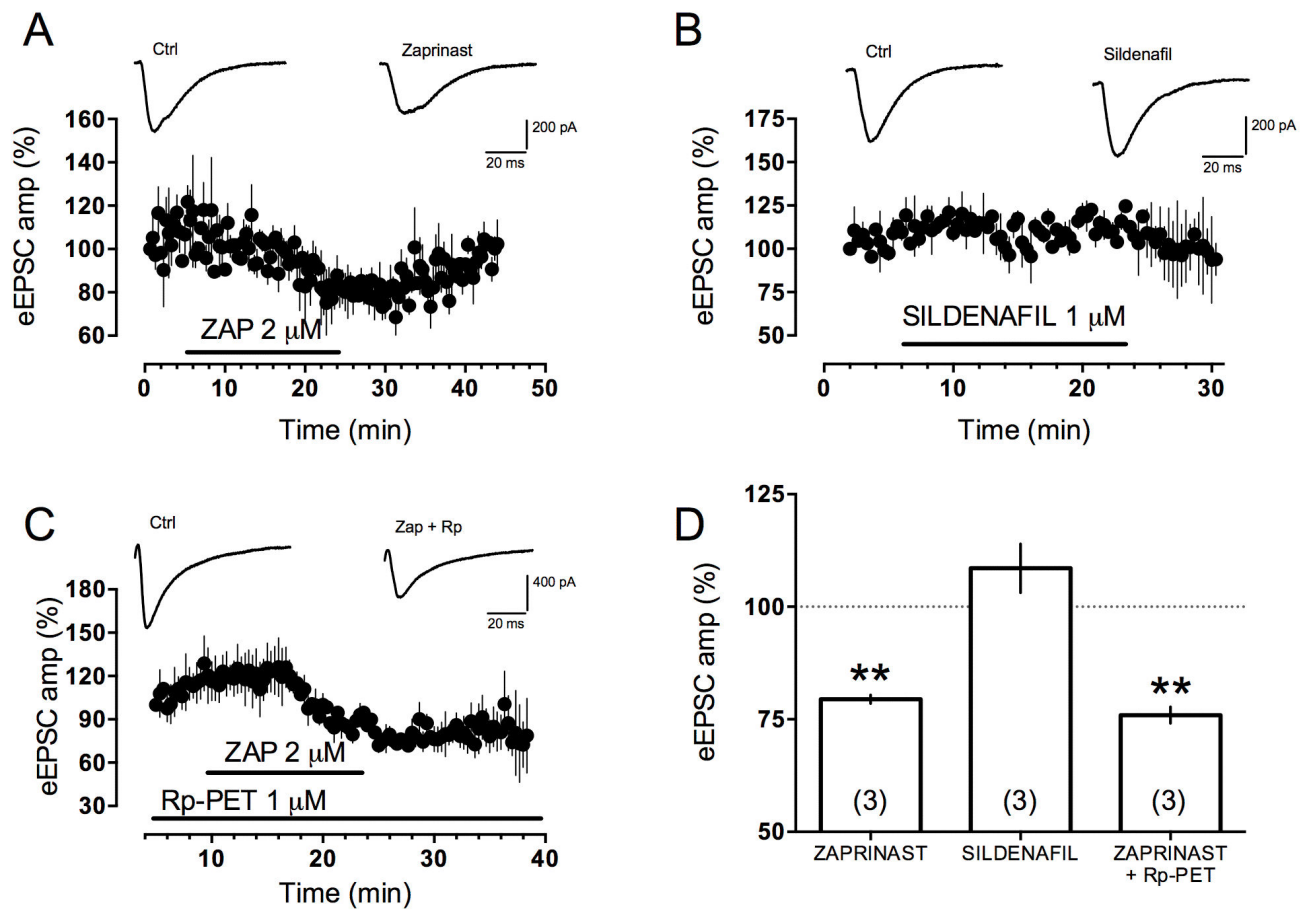
Zaprinast reduces eEPSC amplitude at CA3→CA1 synapse in a PDE5-and cGMP-indipendent manner. **A** and **C**) Time course of the zaprinast-induced decrease in eEPSC amplitude alone (*A*) and in the presence of Rp-8-Br (a cGMP-dependent protein kinase-PKG- inhibitor). EPSCs are normalized to pre-application amplitude values. Sample traces (at *top*) were obtained before (*left*) and during (*right*) zaprinast application and represent averages of 5 traces. Rp-8-Br was pre incubated for at least 10 min before zaprinast application. **B**) Time course of the effect of sildenafil (a PDE5 inhibitor) showing no effect on EPSC amplitude. Sample traces (at *top*) were obtained before (*left*) and during (*right*) sildenafil application and represent averages of 5 traces. **D**) Bar graph of maximal effect of zaprinast application, with or without Rp-8-Br, and sildenafil.

Previous studies demonstrated that KYNA reduces brain extracellular glutamate concentrations and that this action cannot be completely ascribed to NMDA or α7 nicotinic receptors [20]. It has also been reported that KYNA may inhibit excitatory transmission to CA1 pyramidal neurons not only through α7 nicotinic or NMDA receptors, but also through other mechanisms that remain to be identified [35]. In order to study KYNA effects on CA3-CA1 evoked excitatory post synaptic current (eEPSC), KYNA was applied after pharmacological blockade of both α7 nicotinic and NMDA receptors. Under these experimental conditions, KYNA 30 µM still reduced eEPSC suggesting that other mechanisms were involved in this action (Figure 4A black dots and B). Lower KYNA concentrations (1 and 10 µM) were ineffective in this paradigm (data not shown) suggesting a ceiling effect of KYNA 30 µM which does not inhibit AMPA receptor mediated current as previously reported [36]. Therefore, we hypothesized that GPR35 could be involved and to test this proposal, we studied the effects of KYNA in the presence of CID (10 µM), a recently described GPR35 antagonist. CID pre-incubation was able to antagonize KYNA effect on eEPSCs at CA3-CA1 synapse (Figure 4A red dots and B) thus suggesting a GPR35-mediated effect of KYNA in reducing eEPSC amplitude. It is interesting to notice that neither MLA nor APV significantly reduced eEPSC (Figure 4A and B). Moreover, the effects of zaprinast in the same preparation were tested. Zaprinast decreased eEPSCs (Figure 5A) and, in order to rule out the possibility that zaprinast actions were mediated through PDE inhibition and activation of the NO-cGMP- protein kinase (PKG) pathway as previously reported [37,38], we performed experiments in the presence of Rp-8-Br-PET-cGMPS, a PKG inhibitor. Under these experimental conditions, zaprinast still reduced eEPSC amplitude therefore suggesting a mechanism independent from PKG activation (Figure 5C and D). Finally, sildenafil, another potent and selective inhibitor of PDE5, did not modify eEPSC, thus confirming that PDE5 inhibition is not responsible for zaprinast-induced eEPSCs reduction (Figure 5B).

## Discussion

We found that astrocytes express GPR35 transcript and that pharmacological activation of this receptor leads to reduction of i) FRSK-induced cAMP elevation (Figure 1), ii) ATP-induced Ca^2+^ transients (Figure 2) and iii) excitatory synaptic transmission (Figures 4 and 5). Previously, we had reported that basal extracellular glutamate levels were reduced by KYNA in *in vivo* microdialysis experiments [20]. Therefore, it seems reasonable to propose that glial GPR35 could be an important player in the modulation of excitatory synapses. Studies on the molecular mechanism leading from receptor stimulation to dampening of synaptic transmission could therefore be helpful to clarify the role of astrocytes in brain functioning.

As previously mentioned, KYNA is one of the proposed endogenous ligand for GPR35 and zaprinast, a cGMP phosphodiesterase inhibitor, is another potent agonist of this Gi/o coupled receptor [4,5]. In the present study we used both KYNA and zaprinast in order to link the observed effects to GPR35 receptor activation. However, since both compounds are known to interact with a number of other pharmacological targets, particular care was used to rule out the possibility that other receptors or enzymes could be responsible for the observed effects. In fact, beside interacting with GPR35, KYNA is considered an antagonist of the glycine allosteric site the NMDA receptor complex and for several years it was assumed that the interaction between KYNA and the NMDA receptor complex could have a physiological role in brain function [18]. It was then demonstrated that KYNA antagonizes α7 nicotinic receptors that are mostly located on pre-synaptic terminals [19]. KYNA affinity for NMDA or α7 cholinergic nicotinic receptors are rather low (IC50s are: 10-100 µM) and a number of other receptor antagonists are available. In each of our experiments we ruled out the possibility that either α7 nicotine or NMDA receptors could be involved using saturating active concentrations of MLA and D-APV to study the possible involvement of α7 nicotinic or NMDA receptor, respectively. Concerning the second agonist, zaprinast, which is considered the standard reference activator of GPR35 [2,4], since it inhibits PDE (especially 5 and 6), the observation that sildenafil, a specifc PDE5 inhibitor, did not alter eEPSCs amplitude and that Rp-8-Br-PET-cGMPs, a PKG inhibitor, did not prevent the action of zaprinast on eEPSCs, strongly argues in favor of the involvement of GPR35 in zaprinast-dependent depression of eEPSPs. We are therefore rather confident that, in these experimental conditions, the reduction of excitatory synaptic transmission are the consequence of GPR35 activation.

Previous studies reported that a modest increase of KYNA extracellular concentrations in the brain is associated with a number of behavioral effects (reduced locomotor activity, mild analgesia, control of seizures and prevention of excitotoxic neuronal damage) suggesting that KYNA may reduce the activity of brain excitatory transmission at concentrations unable to interact with NMDA receptors [39–42]. It has also been demonstrated that a two-threefold elevation of brain KYNA levels significantly reduces post-ischemic brain damage in models of focal or global brain ischemia *in vivo* and in organotypic hippocampal slice cultures exposed to oxygen and glucose deprivation *in vitro* [43]. An increase of brain KYNA levels may be obtained by administering direct or indirect precursors, transport inhibitors or inhibitors of kynurenine 3-monooxygenase (KMO) the most abundant of the kynurenine metabolizing enzymes. No matter of the approach used, a mild increase of brain KYNA concentration reduces excitatory transmission and this may be evaluated with biochemical, electrophysiological, histological or behavioral methods [44,45]. Recently, a very elegant study reported that inhibition of kynurenine 3-monooxygenase in peripheral organs, by increasing blood kynurenine levels and brain KYNA content, significantly reduced neurodegeneration in different transgenic models of Huntington’s and Alzheimer’s diseases [46]. Similar results have been obtained in a drosophila model of Huntington chorea [47]. KYNA is mostly formed in astrocytes [48] and a local increase of its extracellular concentration drastically reduces extracellular brain glutamate content [20,49], although in different brain areas. A reduction of astrocytic glutamate output could contribute to the robust decrease of excitatory transmitter levels in brain extracellular spaces associated with increased KYNA synthesis [20] and could explain most of behavioral, electrophysiological and neuroprotective effects of KYNA. The molecular mechanisms leading from KYNA-induced activation of GPR35 to the reduced glutamate output from astrocytes could well include a reduced cAMP formation and a decrease of Ca^2+^ transients (Figure 2). While the molecular mechanism leading from the stimulation of a receptor coupled to Gi/o to a decrease cAMP synthesis have been widely investigated and are sufficiently elucidated, the mechanisms leading to changes in the Ca^2+^ transients and reduced transmitter output remain to be clarified. A number of possible cross-talk pathway between cAMP and Ca^2+^ signaling have been described and could be operative in our model [50,51]. In astrocytes, in particular, it was proposed that changes in cellular cAMP concentration may modulate CCE [22]. Therefore, we focused our attention on this current and we found that the actions of either KYNA or zaprinast on the plateau phase of ATP-induced Ca^2+^ transients were qualitatively similar to those of MRS 1845 a selective inhibitor of the store operated channels which are responsible for this current. When SOCs were studied with a specific protocol (see Figure 3) we noticed that the action of KYNA and those of MRS could be easily differentiated, thus ruling out the possibility that the effects of GPR35 activation on the plateau phase of ATP-induced calcium transients could be ascribed to changes of the capacitative calcium entry. The effects of GPR35 activation on Ca^2+^ transients remain therefore to be clarified. In excitable cells, such as rat sympathetic neurons, expressing transfected GPR35, it has been shown that GPR35 agonists modulate the voltage operated calcium channels of the N-type which are obviously involved in controlling transmitter release [52]. In astrocytes the N-type channels are not expressed and the mechanism controlling the Ca^2+^ sources involved in exocytotic glutamate release remain to be clarified [53]. It is clear, however, that GPR35 agonists modulate astrocytic Ca^2+^ fluxes, transmitter release and synaptic transmission. It is also well demonstrated that by selectively stimulating nearby astrocytes it is possible to modify synaptic currents and regulate neuronal plasticity [54]. Our data, by demonstrating that both KYNA and zaprinast decrease excitatory synaptic currents strongly suggest that astrocytic receptors may control the levels of synaptic transmitter and the function of neuronal circuits. GPR35 mediated effects were studied both in mouse and rat and the overall results suggest a KYNA-induced GPR35 effect in both species. KYNA GPR35 mediated effects on eEPSCs were tested in rats were KYNA actions on synaptic transmission were better characterized [35]. Finally, our observation suggest that GPR35 may be an interesting pharmacological target since GPR35 agonists could have a role in decreasing pain transmission [29] and reducing excitotoxic damage in a number of clinical conditions [6,7,11].
