# Correction: GPR35 Activation Reduces Ca^2+^ Transients and Contributes to the Kynurenic Acid-Dependent Reduction of Synaptic Activity at CA3-CA1 Synapses

**DOI:** 10.1371/annotation/1a731868-9fe9-4e52-9e45-5b11f6fbcfdb

**Published:** 2014-01-02

**Authors:** Rolando Berlinguer-Palmini, Alessio Masi, Roberto Narducci, Leonardo Cavone, Dario Maratea, Andrea Cozzi, Maria Sili, Flavio Moroni, Guido Mannaioni

The first two authors, Rolando Berlinguer-Palmini and Alessio Masi, should be indicated as having contributed equally to the article. 

